# Targeting Phosphodiesterase 4 in Gastrointestinal and Liver Diseases: From Isoform-Specific Mechanisms to Precision Therapeutics

**DOI:** 10.3390/biomedicines13061285

**Published:** 2025-05-23

**Authors:** Can Chen, Mei Liu, Xiang Tao

**Affiliations:** 1 Institute of Liver and Gastrointestinal Diseases, Department of Gastroenterology, Tongji Hospital, Tongji Medical College, Huazhong University of Science and Technology, Wuhan 430030, China; 2 Clinical Center of Human Gene Research, Union Hospital, Tongji Medical College, Huazhong University of Science and Technology, Wuhan 430022, China

**Keywords:** phosphodiesterase 4 inhibitors, inflammatory bowel disease, liver diseases, gastrointestinal disorders, drug development, personalized medicine

## Abstract

Phosphodiesterase 4 (PDE4) serves as a crucial regulator of cyclic adenosine monophosphate (cAMP) signaling and has been identified as a significant therapeutic target for inflammatory and metabolic disorders impacting the gastrointestinal (GI) tract and liver. Although pan-PDE4 inhibitors hold therapeutic promise, their clinical use has been constrained by dose-dependent adverse effects. Recent progress in the development of isoform-specific PDE4 inhibitors, such as those selective for PDE4B/D, alongside targeted delivery systems like liver-targeting nanoparticles and probiotic-derived vesicles, is reshaping the therapeutic landscape. This review consolidates the latest insights into PDE4 biology, highlighting how the structural characterization of isoforms informs drug design. We conduct a critical evaluation of preclinical and clinical data across various diseases, including inflammatory bowel diseases (IBDs), alcoholic liver disease, nonalcoholic fatty liver disease (NAFLD), liver fibrosis, and digestive tract tumors, with an emphasis on mechanisms extending beyond cAMP modulation, such as microbiota remodeling and immune reprogramming. Additionally, we address challenges in clinical translation, including biomarker discovery and the heterogeneity of trial outcomes, and propose a roadmap for future research directions.

## 1. Introduction

Cyclic nucleotide phosphodiesterases (PDEs) are a superfamily of enzymes that regulate the spatial and temporal dynamics of second messenger signaling within the cellular system. Among the 11 distinct families of PDEs, phosphodiesterase 4 (PDE4) stands out as the largest cyclic adenosine monophosphate (cAMP)-specific family, thereby influencing numerous biological processes in both health and disease [1,2]. The PDE4 family encompasses four subtypes, PDE4A, PDE4B, PDE4C, and PDE4D, which are differentially expressed across various tissues, including the cardiovascular system, central nervous system, and other organs [3,4,5,6]. This subtype diversity is further amplified by alternative splicing, generating over 20 isoforms that achieve functional specialization through structural features such as upstream conserved regions (UCRs). These domains govern dimerization, subcellular compartmentalization, and interactions with scaffolding proteins, enabling precise modulation of cAMP gradients within localized microdomains. Given its central role in cAMP metabolism, PDE4 has emerged as a compelling therapeutic target. Pan-PDE4 inhibitors enhance cAMP signaling by broadly blocking all subtypes, demonstrating efficacy in inflammatory diseases such as psoriasis and chronic obstructive pulmonary disease (COPD). However, the clinical application of PDE4 inhibitors has been limited due to adverse side effects associated with non-selective inhibition of all PDE4 isoforms [7]. Recent advances in structural biology and subtype-selective inhibitor design have begun to disentangle the therapeutic potential of individual PDE4 subtypes. Understanding the distinct roles of PDE4 isoforms and their specific contributions to disease pathology has opened up new avenues for developing selective inhibitors that can maximize therapeutic benefits while minimizing side effects [5,7]. Despite these breakthroughs, systematic evaluations of their therapeutic potential in gastrointestinal (GI) and hepatic pathologies are lacking.

This comprehensive review presents current research on PDE4 and its potential utility as a therapeutic target in GI and liver diseases, including inflammatory bowel diseases (IBDs), alcoholic liver disease (ALD), nonalcoholic fatty liver disease (NAFLD), liver fibrosis, digestive tract tumors, and possibly other conditions. By integrating preclinical and clinical data, we propose that subtype-selective PDE4 inhibition could revolutionize the management of GI and hepatic disorders while circumventing historical toxicity challenges. Furthermore, we discuss innovative strategies such as tissue-targeted drug delivery and allosteric modulation to optimize therapeutic outcomes.

## 2. Structure, Gene Variants, and Properties of Phosphodiesterase 4

PDE4 is a critical enzyme in the regulation of cAMP levels within cells, influencing various physiological processes and signaling pathways. The PDE4 family consists of four main isoforms: PDE4A, PDE4B, PDE4C, and PDE4D, each of which can be further divided into multiple gene variants (>25) due to alternative splicing. This diversity in isoforms and variants allows for a nuanced regulation of cAMP signaling, tailored to specific cellular contexts and stimuli [8,9,10].

The various PDE4 isoforms can be classified into three distinct subtypes based on their structural characteristics. The long forms contain two UCRs, UCR1 and UCR2, located in the N-terminal region. In contrast, the short forms retain only the UCR2, while the super-short forms lack UCR1 and possess a truncated UCR2 [8,11]. Additionally, there exist dead-short forms of PDE4, which are less well-characterized and are believed to exhibit minimal or no catalytic activity. These isoforms play an important role in modulating signaling pathways indirectly, possibly by sequestering cAMP or interacting with other proteins [11].

**Figure 1:**
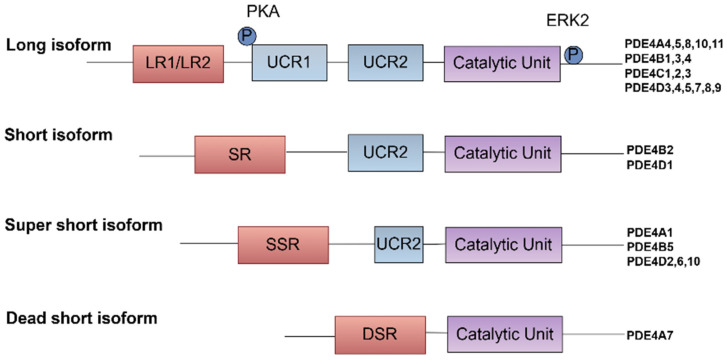
Classification and post-translational regulation of PDE4 isoforms. PDE4 isoforms are classified based on the presence of UCRs, which are critical for their structural and functional diversity. The UCRs, specifically UCR1 and UCR2, are crucial for controlling the catalytic activity of PDE4 isoforms and their interactions with other proteins, which can influence their localization and function within the cell.

These UCRs are significant for regulating the enzyme’s activity and its interaction with other proteins [7,9,12,13]. The dimerization of PDE4, facilitated by interfaces located in both the UCR1 and catalytic unit domains, is essential for the enzyme’s stability and activity, underscoring the importance of these conserved regions in maintaining the functional integrity of PDE4 enzymes [14]. Furthermore, the UCRs contribute to the specificity of PDE4’s interactions with other proteins and signaling molecules, enabling compartmentalized cAMP signaling. This precise regulation allows for the fine-tuning of cellular responses to external stimuli [15]. Notably, the UCR1 region is subject to phosphorylation by protein kinase A (PKA), which enhances enzyme activity and provides precise control over intracellular cAMP levels [16]. Additionally, the phosphorylation of PDE4 by ERK can modulate its activity, affecting its ability to hydrolyze cAMP and thus influencing the intensity and duration of cAMP-mediated signaling pathways [7] (Figure 1).

Moreover, the spatial and temporal compartmentalization of cAMP signaling, regulated by PDE4, is crucial for the specificity of cellular responses. This compartmentalization allows for localized signaling events that can lead to different physiological outcomes depending on the cellular context. For instance, in cardiac myocytes, cAMP signaling is organized into nanodomains that facilitate precise regulation of heart function, allowing for tailored responses to sympathetic stimulation [17,18]. Therefore, a deeper understanding of PDE4 isoform-specific functions and their interactions with other signaling pathways is essential for the development of targeted therapies aimed at enhancing cAMP signaling in various pathological conditions.

The development of selective PDE4 inhibitors has garnered significant attention due to their potential to enhance cAMP signaling while minimizing adverse effects associated with broader PDE inhibition [19]. Grasping the structure–function connections of PDE4 isoforms is crucial for developing new therapeutic drugs. Advances in structural biology, particularly through X-ray crystallography, have provided valuable insights into the molecular mechanisms underlying the regulation and inhibition PDE4 [20]. These findings pave the way for the development of more effective and selective PDE4 inhibitors that could be employed across the treatment of various diseases, including COPD, depression, and inflammatory disorders [21].

## 3. Integrated Signaling Network of PDE4-cAMP Axis in GI and Liver Diseases

Ligand interaction with G protein-coupled receptors (GPCRs) initiates cAMP synthesis, primarily catalyzed by adenylate cyclases (ACs), and cAMP synthesis is dynamically hydrolyzed by PDE4 isoforms to fine-tune downstream signaling pathways. The phosphorylation of PDE4 by PKA or EKR was reported to modulate enzyme activity and subcellular localization and provide precise control over intracellular cAMP levels [7,16]. Inhibition of PDE4 has emerged as a promising therapeutic strategy for various diseases due to its ability to elevate intracellular cAMP levels, exerting its effects through various effector proteins, including PKA, exchange protein directly activated by cAMP (EPAC), and others like Popeye domain containing (POPDC) and hyperpolarizing cyclic nucleotide-gated (HCN) channels [22,23,24].

Among these, PKA and EPAC are the most studied, each mediating distinct pathways and cellular responses. PDE4 inhibitors have shown potential in treating ulcerative colitis (UC) by modulating the cAMP/PKA/CREB and EPAC-Rap1 signaling pathway, which contributed to the suppression of pro-inflammatory pathways such as MAPK, NF-κB, PI3K-mTOR, and JAK-STAT-SOCS3 [25]. This modulation helps reduce inflammation and restore mucosal homeostasis, highlighting the therapeutic potential of PDE4 inhibition in managing chronic inflammatory conditions [26]. PDE4-cAMP signaling can also modulate chloride secretion via cystic fibrosis transmembrane conductance regulator (CFTR) [27,28]. Moreover, roflumilast, a selective PDE4 inhibitor, has been shown to have significant therapeutic potential in the treatment of polymicrobial sepsis-induced liver damage by inhibiting key inflammatory pathways, including the NF-κB, p38 MAPK, and STAT3 pathways [29]. In addition, PDE4-cAMP signaling intersects with metabolic and fibrotic pathways in the liver. One study highlights the role of oleic acid in stimulating the cAMP/PKA pathway, which subsequently activates peroxisome proliferator-activated receptor alpha (PPARα) to enhance fatty acid (FA) β-oxidation [30]. EPAC proteins, through their regulation of calcium handling, may influence the progression of liver disease by modulating endoplasmic reticulum (ER) stress responses. The ER is a major site for protein and lipid synthesis and calcium storage, and disturbances in ER homeostasis can lead to stress and activation of the unfolded protein response. Chronic ER stress is known to play a role in the development of insulin resistance and diabetes, conditions often associated with obesity and liver disease. Alterations in lipid and calcium metabolism within the ER can exacerbate these conditions, suggesting that EPAC-mediated pathways could be crucial in managing hepatic ER stress and improving glucose homeostasis [31,32]. It is important to mention that PDE4 is significantly expressed in activated HSCs [33]. The inhibition of PDE4 not only facilitates the dedifferentiation of myofibroblasts but also mitigates the production of reactive oxygen species (ROS) and the onset of fibroblast senescence, both of which are induced by transforming growth factor-beta 1 (TGF-β1) [34]. These effects are crucial for reversing liver fibrosis and restoring normal tissue architecture, offering hope for improved treatment options in conditions characterized by excessive fibrotic tissue remodeling. Moreover, PKA and EPAC play significant roles in activating endothelial nitric oxide synthase (eNOS), which is crucial for the regulation of various liver diseases. The activation of eNOS by these cAMP effectors involves complex signaling pathways that enhance nitric oxide (NO) production, as demonstrated in studies using human umbilical vein endothelial cells (HUVECs) [35,36]. In HCC, PDE4D interacts with yes-associated protein (YAP) to enhance tumor growth. Inhibition of PDE4 with roflumilast resulted in cAMP-PKA activation and YAP phosphorylation, limiting hepatocellular carcinoma (HCC) cell growth [37].

**Figure 2:**
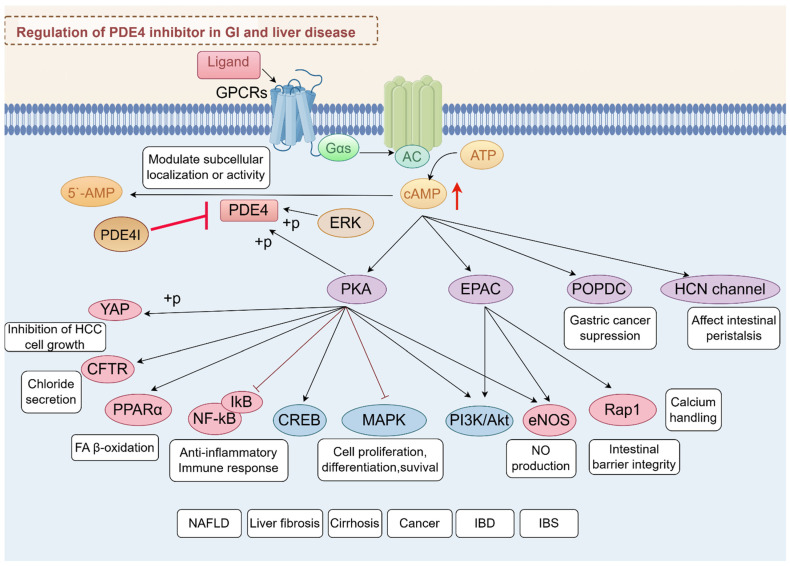
Integrated signaling network of PDE4-cAMP axis in GI and liver diseases. This schematic summarizes the regulatory roles of PDE4 in modulating cAMP-dependent pathways and their downstream effects across digestive system pathologies.

POPDC proteins, comprising POPDC1, POPDC2, and POPDC3, play roles in numerous cellular functions, such as cell signaling and membrane trafficking. They are considered potential therapeutic targets in gastric cancer (GC) due to their role in cancer cell behavior [38,39]. In the context of the GI system, HCN4 channels are expressed in specific neurons that contribute to the regulation of peristaltic movements, including retrograde peristalsis [40]. Above all, the PDE4-cAMP axis serves as a central regulatory hub in GI and liver diseases (Figure 2), orchestrating diverse cellular processes through compartmentalized signaling networks.

## 4. Preclinical Evidence of PDE4 Inhibitors in GI and Liver Disease

### 4.1. IBD

IBD encompasses a group of chronic inflammatory conditions that primarily affect the GI tract, notably including Crohn’s disease (CD) and UC. The pathogenesis of IBD is complex and results from a multifactorial interplay among genetic, environmental, and immunological factors, which collectively contribute to chronic inflammation and damage to the intestinal mucosa [41,42].

Research indicates that PDE4A, PDE4B, and PDE4D are upregulated in the colon in UC models. Elevated PDE4 activity is associated with increased gut inflammation in chronic UC patients, and targeting this enzyme with apremilast may offer a therapeutic strategy to alleviate clinical symptoms of chronic UC by reducing mucosal ulcerations and inflammatory infiltrates in a murine chronic UC model induced by dextran sulfate sodium (DSS) [25]. Furthermore, the therapeutic effects of PDE4 inhibition extend beyond symptom relief; it has been observed that these inhibitors can also remap the gut microbiota, enhancing intestinal barrier function and restoring microbial balance. This suggests that targeting PDE4 may not only address the inflammatory aspects of UC but also contribute to the overall health of the gut microbiome [26]. The inhibition of PDE4B has been linked to the upregulation of anti-inflammatory pathways through the regulation of CYLD, indicating a potential mechanism by which these inhibitors exert their beneficial effects. This underscores the importance of PDE4 as a therapeutic target in the management of chronic UC and suggests that further research into PDE4 inhibitors could lead to improved treatment strategies for patients suffering from this debilitating condition [43]. The role of microRNAs (miRNAs) in modulating inflammatory responses has garnered significant attention in recent years, particularly in the context of intestinal inflammation. One such miRNA, miR-369-3p, has been implicated in regulating of inflammatory pathways in IBD by targeting PDE4B [44].

Natural compound-derived PDE4 inhibitors are emerging due to their favorable safety profiles and potent efficacy in models of IBD. For instance, mangostanin has been developed, demonstrating both favorable safety profiles and potent anti-inflammatory effects in IBD models [45]. The exploration of new PDE4 inhibitors is critical, as traditional therapies often come with significant side effects. For example, the discovery of N2-indazole derivatives (LZ-14) has shown promise in selectively inhibiting PDE4D7, thus potentially enhancing therapeutic outcomes while minimizing adverse effects [46]. Fucoidan, a sulfated polysaccharide derived from brown seaweeds, has also received considerable attention in recent years, particularly due to its favorable safety profiles in IBD models. This effect is largely attributed to its ability to modulate the aryl hydrocarbon receptor (AHR) and PDE4 [47]. Research indicates that fucoidan can modulate gut microbiota, enhance the abundance of beneficial bacteria, and reduce levels of pro-inflammatory cytokines, positioning it as a promising candidate for the management of IBD symptoms and progression. In addition to its immunomodulatory effects, fucoidan has been investigated for its ability to restore gut microbiota diversity, which is often disrupted in patients with IBD. A study demonstrated that treatment with fucoidan significantly alleviated symptoms of IBD in a DSS-induced mouse model, underscoring its potential as a gut microbiota modulator. Furthermore, fucoidan’s ability to elevate levels of short-chain FAs, which benefit gut health, further supports its role in promoting a balanced gut microbiome [48]. The compound Hypersampsonone H (HS-1), isolated from the plant species Hypericum sampsonii Hance, has shown promising effects in the treatment of UC. In vitro studies indicated that HS-1 effectively reduced lipopolysaccharide (LPS)-induced inflammatory responses in RAW264.7 cells. This was evidenced by a decrease in the production of NO and the downregulation of pro-inflammatory cytokines such as IL-6 and TNF-α. Furthermore, HS-1 was found to inhibit the expression of cyclooxygenase-2 (COX-2) and inducible nitric oxide synthase (iNOS), which are critical mediators in the inflammatory process. Treatment with HS-1 resulted in significant improvements in DSS-induced colitis, including reduced weight loss and colon length, which are indicative of colonic inflammation. Histopathological analyses revealed that HS-1 treatment preserved the integrity of the intestinal epithelial barrier and reduced tissue fibrosis, highlighting its protective effects against colonic damage by regulating the PDE4 signaling pathway, leading to increased intracellular levels of cAMP and subsequent activation of PKA and CREB signaling pathways [49].

Moreover, a recent study presented a novel approach to treating UC by utilizing a dual-drug delivery system. This system employs membrane vesicles derived from the probiotic Escherichia coli Nissle 1917 (EcN 1917) to target macrophages, which play a crucial role in the inflammatory processes associated with UC. The combination of roflumilast, a PDE4 inhibitor, and manganese dioxide (MnO_2_) aims to enhance the levels of cAMP within macrophages, thereby modulating their inflammatory response and reducing the production of pro-inflammatory cytokines such as TNF-α. This innovative strategy not only addresses the immediate inflammatory response but also seeks to restore a healthier gut microbiome by mitigating pathogenic bacteria while promoting beneficial strains like Akkermansia [50].

In summary, PDE4 represents a promising target for therapeutic intervention in IBD [51,52,53], with potential implications for both GI and liver health. Continued exploration of PDE4 inhibitors and their effects on the immune system may lead to more effective treatments for patients suffering from these debilitating conditions. Moreover, the specificity of PDE4 inhibitors for different isoforms of the enzyme may enhance their therapeutic potential, allowing for targeted treatment that minimizes adverse effects commonly associated with broader anti-inflammatory therapies. As research continues to elucidate the complex role of PDE4 in inflammation, it is becoming increasingly clear that these inhibitors could play a significant role in the management of IBD and other chronic inflammatory diseases. The ongoing research into PDE4 inhibitors not only underscores their potential as effective treatments for IBD but also opens avenues for addressing other GI and liver diseases.

### 4.2. NAFLD

NAFLD represents a significant global health concern, characterized by a spectrum of liver abnormalities that range from simple steatosis (nonalcoholic fatty liver, NAFL) to more severe forms such as nonalcoholic steatohepatitis (NASH), which can lead to cirrhosis and HCC. The prevalence of NAFLD has been rising in parallel with the global increase in obesity and metabolic syndrome, affecting approximately 25% of the population worldwide. This condition is not only a leading cause of chronic liver disease but is also associated with increased risks of cardiovascular disease and other metabolic complications, making it a critical area of research and clinical focus [54,55].

PDE4 has emerged as a significant player in the pathogenesis of NAFLD [56,57]. The role of PDE4 in NAFLD is multifaceted, involving the modulation of inflammatory pathways and lipid metabolism, which are critical in the disease’s progression and associated complications. Roflumilast, a PDE4 inhibitor, has been shown to exert beneficial effects on liver health by influencing various metabolic and inflammatory processes. Recent studies have highlighted its potential in ameliorating hepatic steatosis and inflammation, thereby addressing key components of NAFLD pathology [58,59,60]. The overexpression of PDE4D in the liver has been shown to contribute to the development of NAFLD and hypertension through the activation of FA translocase CD36 signaling, which facilitates lipid deposition in hepatocytes and promotes the secretion of TGF-β1, a cytokine that plays a pivotal role in fibrosis and inflammation. Furthermore, the PDE4 inhibitor roflumilast has demonstrated efficacy in reducing hepatic steatosis and improving liver function in mouse models [61].

Recent studies have highlighted the potential of 2-(4-([2-(5-Chlorothiophen-2-yl)-5-ethyl-6-methylpyrimidin-4-yl] amino) phenyl) acetic acid (A-33) in ameliorating chronic liver disease (CLD) and metabolic disorders. A-33, a PDE4B inhibitor, has shown promising results in mice models, particularly in the context of steatotic liver disease. The compound effectively reduced serum alanine aminotransferase (ALT) and aspartate aminotransferase (AST) levels, indicating a decrease in liver injury. Furthermore, A-33 was associated with reduced fat and collagen deposition in the liver, normalization of intrahepatic triglyceride concentrations, and modulation of gene expression related to FA β-oxidation and inflammation. In addition to A-33, a novel analog known as MDL3 has been synthesized, and it inhibits both PDE4B and PDE5A. This compound demonstrated superior efficacy in ameliorating the pathophysiological signs of liver injury compared to A-33. Notably, MDL3 resensitized obese mice to glucose and inhibited pathological remodeling of adipose tissue, which was not observed with A-33 administration. These findings suggest that MDL3 may represent a more effective therapeutic option for treating CLD and associated metabolic disorders [62].

The systemic implications of NAFLD, including its association with cardiovascular disease and other metabolic disorders, underscore the importance of understanding the role of PDE4 in this context. NAFLD is not merely a hepatic condition; it is associated with increased risks of cardiovascular events and other extrahepatic complications [63,64,65]. The mechanisms linking NAFLD to these systemic outcomes are still being elucidated, but the involvement of inflammatory mediators and metabolic dysregulation is evident.

The development of ASP9831, a PDE4 inhibitor aimed at treating NASH, highlights the challenges faced in translating preclinical success into clinical efficacy. In preclinical studies, ASP9831 demonstrated potent anti-inflammatory and antifibrotic effects, leading to optimism regarding its potential therapeutic benefits. Despite the promising preclinical data, the results from the phase 1 and 2 trials indicated that ASP9831 did not significantly alter the biochemical markers of NASH when compared to placebo. Specifically, there were no significant changes observed in mean serum levels of ALT or AST across the treatment groups. Most adverse events reported were mild, with GI disorders occurring more frequently in the ASP9831 groups than in the placebo group. These findings underscore the challenges faced in developing effective therapeutics for NASH and highlight the need for more extensive preclinical testing of potential drug candidates before advancing to clinical trials [66,67]. The ineffectiveness of ASP9831 in NASH treatment likely stems from a combination of factors, including its limited mechanistic scope, suboptimal trial design, patient-selection issues, pharmacokinetic variability, biomarker inadequacies, and the inherent complexity of NASH. Future research should adopt a more comprehensive strategy, incorporating innovative biomarkers, longer trial durations, and potentially combination therapies to enhance treatment outcomes for NASH patients.

In conclusion, PDE4 represents a promising target for therapeutic intervention in NAFLD, given its central role in both inflammatory and metabolic pathways. Continued research into the precise mechanisms by which PDE4 contributes to the pathogenesis of NAFLD could lead to the development of novel treatment strategies aimed at mitigating liver injury and its associated systemic effects. The integration of PDE4 inhibitors into clinical practice may provide a multifaceted approach to managing NAFLD and its complications, ultimately improving patient outcomes.

### 4.3. ALD

ALD is a significant contributor to liver-related mortality worldwide, with its prevalence increasing due to rising alcohol consumption and associated risk factors. The complexity of ALD encompasses various stages, including fatty liver, alcoholic hepatitis, and cirrhosis, each presenting unique challenges for diagnosis and treatment. Recent research has underscored the multifaceted nature of ALD, emphasizing the role of oxidative stress, inflammation, and gut microbiome interactions in its pathogenesis [68,69,70,71].

PDE4 has gained significant attention as a pivotal pathway in the pathobiology of ALD. Chronic alcohol consumption is known to induce an upregulation of various isoforms of PDE4, specifically PDE4A, PDE4B, and PDE4D, which play crucial roles in modulating cAMP signaling within hepatic tissues. This dysregulation of cAMP levels is associated with impaired lipid metabolism and inflammatory responses, contributing to the progression of liver injury and steatosis in the context of alcohol exposure [72,73,74]. Exploration of PDE4 inhibitors like roflumilast and A-33 has uncovered their therapeutic potential in lessening alcohol-induced liver fat and inflammation through various mechanisms, including the modulation of oxidative stress and the restoration of normal lipid metabolism [62,73]. Specifically, by augmenting LPS-inducible TNF expression, PDE4B serves as a crucial mediator in the pathophysiology of ALD, positioning it as a potential therapeutic target aimed at reducing inflammation and improving liver health in individuals with alcohol-use disorders (AUDs) [75]. The selective deletion of PDE4B or rolipram treatment has been highlighted as a promising therapeutic strategy, as it not only reduces inflammatory cytokine production but also enhances the metabolic functions of liver cells, thereby mitigating the adverse effects of alcohol on liver health [72]. Recent studies have highlighted the potential of novel inhibitors, such as MDL3, which targets both PDE4B and PDE5A, in mitigating the effects of ALD by enhancing cAMP signaling pathways. The dual inhibition of PDE4B and PDE5A by MDL3 may offer a more favorable therapeutic profile, potentially minimizing adverse effects while maximizing therapeutic benefits [62].

Apremilast, a selective PDE4 inhibitor, has been primarily utilized in the treatment of psoriasis and psoriatic arthritis. However, emerging evidence suggests its potential in reducing alcohol consumption, thereby addressing a critical public health issue associated with alcohol-use disorders. Preclinical research has revealed that apremilast can effectively reduce binge-like alcohol intake and influence behavioral measures of alcohol motivation across different animal models, including those genetically predisposed to excessive drinking. In addition to its effects described in animal models, clinical investigations have also highlighted the efficacy of apremilast in human subjects. The Phase IIa research indicated that apremilast substantially decreased excessive alcohol intake in non-treatment-seeking individuals with AUD, showcasing its potential as a therapeutic option for this population. It appears that the core mechanisms involve changes in neural activity within the nucleus accumbens, an essential brain region linked to the regulation of alcohol use. This neurobiological insight underscores the relevance of apremilast not only in treating AUD but also in understanding the complex interplay between pharmacotherapy and neural circuitry associated with addiction [76].

ZL40, identified as a novel PDE4 inhibitor, exhibited potential in alleviating alcoholic liver damage and reducing inflammation in the NIAAA mice model, making it a promising candidate for ALD treatment, which is characterized by oxidative stress, inflammation, and liver damage due to excessive alcohol consumption. The underlying mechanisms of ZL40’s protective effects may involve modulation of inflammatory pathways and enhancement of liver function, similar to findings observed with other therapeutic agents [77].

One significant study demonstrated that the PDE4 inhibitor rolipram, when delivered via a targeted nanoparticle system, effectively reduced hepatic steatosis and injury in models of ALD. This delivery method not only improved the bioavailability of the drug but also localized its action to the liver, thereby avoiding the central nervous system side effects typically associated with PDE4 inhibitors [73]. KVA-D88, a novel PDE4B inhibitor, has shown promising results in preclinical studies, particularly in mitigating the inflammatory responses associated with ALD. The encapsulation of KVA-D88 into mPEG-b-P(CB-co-LA) nanoparticles not only enhances the solubility and bioavailability of the drug but also improves its therapeutic efficacy compared to the free drug form. In vivo studies demonstrated that KVA-D88-loaded NPs significantly ameliorated alcohol-induced hepatic injury and inflammation, showcasing their potential as a more effective treatment option for ALD [78].

### 4.4. Liver Fibrosis and Cirrhosis

Liver fibrosis, characterized by the excessive accumulation of extracellular matrix (ECM) components in response to chronic liver injury, represents a significant health concern globally. This pathological process is often driven by various underlying conditions, including viral hepatitis, ALD, and NAFLD [79,80]. The activation of hepatic stellate cells (HSCs) plays a pivotal role in the development of liver fibrosis, as these cells transdifferentiate from a quiescent state into proliferative fibrogenic myofibroblasts, contributing to the fibrotic response [81,82]. The accumulation of ECM not only disrupts the normal architecture of the liver but also leads to significant complications, including liver cirrhosis, portal hypertension, and, ultimately, liver failure. Liver cirrhosis, characterized by the progressive replacement of healthy liver tissue with scar tissue, is often a consequence of chronic liver disease. This condition arises from various etiologies, including viral hepatitis, alcohol abuse, and NAFLD. The pathophysiology of cirrhosis involves a complex interplay of inflammation, fibrogenesis, and regenerative processes. Over time, the liver’s architecture becomes distorted, leading to impaired function and the development of complications such as portal hypertension and liver failure [83,84].

Recent studies have highlighted that PDE4 expression is markedly increased in activated HSCs, correlating with the fibrogenic process observed in various liver diseases, including NASH and cirrhosis. Targeted delivery of rolipram to the liver has been shown to prevent fibrogenesis effectively. This targeted approach not only enhances the therapeutic efficacy of rolipram but also minimizes systemic side effects, making it a viable option for clinical application in liver fibrosis management [33]. The specific PDE4 inhibitor rolipram has also been shown to significantly attenuate liver injury and fibrosis in bile duct ligation models in rats. This effect is attributed to the reduction of inflammatory cytokine expression and the inhibition of HSC activation, which are critical processes in the development of liver fibrosis [85]. In a study focusing on the renal responses of cirrhotic rats, it was observed that rolipram infusion led to a notable increase in the excretion of sodium and phosphate. This finding is particularly relevant given the challenges associated with managing fluid retention and electrolyte balance in patients with liver cirrhosis, a condition often complicated by ascites [86]. Furthermore, the modulation of inflammatory pathways through PDE4 inhibition with roflumilast has been linked to improved liver function and reduced fibrosis markers in experimental models induced by diethylnitrosamine (DEN) and GAN diet [60,87] (Table 1). Recent findings have highlighted a previously unrecognized anti-fibrotic pathway where Smurf2 interacts with PDE4B, leading to the degradation of PDE4B and attenuation of liver fibrosis. This interaction is crucial, as it underscores the potential of targeting Smurf2 and its associated PDE4B for therapeutic interventions in fibrotic diseases [88].

In conclusion, the role of PDE4 in liver fibrosis is multifaceted, involving direct effects on HSC activation and indirect modulation of inflammatory pathways. As research continues to elucidate the intricate mechanisms underlying liver fibrosis, targeting PDE4 presents a promising strategy for therapeutic intervention. Future studies should focus on the clinical translation of PDE4 inhibitors and their potential to reverse or halt the progression of liver fibrosis, ultimately improving patient outcomes.

### 4.5. Cancer

Recent studies have highlighted the dual role of cAMP in cancer, where it can either promote or inhibit tumor growth depending on the specific cellular context and the type of cancer involved. For instance, in some cases, cAMP signaling through PKA has been shown to inhibit cell proliferation and induce apoptosis, acting as a tumor suppressor. Conversely, in other contexts, cAMP can activate pathways that promote tumor growth and metastasis, particularly through the activation of EPAC and its downstream effectors, such as Rap1 and other small GTPases. This complexity underscores the necessity of a nuanced understanding of cAMP signaling in cancer, as it may provide insights into the mechanisms of tumorigenesis and potential therapeutic targets [95]. PDE4 inhibitors have emerged as promising therapeutic agents in cancer treatment due to their ability to elevate cAMP levels, a second messenger that influences numerous signaling pathways involved in cell proliferation, apoptosis, and inflammation [7,96]. Research indicates that PDE4 inhibition can downregulate B-cell receptor (BCR)-related kinases, thereby inducing apoptosis in cancer cells and blocking angiogenesis within the tumor microenvironment (TME). This suggests that PDE4 inhibitors not only enhance cAMP signaling but also provide a mechanism to counteract the immunosuppressive environment often found in tumors [97].

#### 4.5.1. HCC

HCC is recognized as the most prevalent form of primary liver cancer, presenting significant challenges in treatment due to its aggressive nature and the limited efficacy of existing therapeutic modalities. The incidence of HCC has been steadily rising, making it a major global health concern, with an estimated 850,000 new cases diagnosed annually [98]. Risk factors for HCC include chronic infections with hepatitis B and C viruses, alcohol consumption, and, increasingly, NAFLD [54,99]. The prognosis for patients diagnosed with HCC remains poor, primarily because many cases are detected at advanced stages, when curative options are no longer viable [100]. Current treatment strategies for HCC include surgical resection, liver transplantation, and locoregional therapies such as transarterial chemoembolization (TACE). Surgical resection is often the first-line treatment for patients with early-stage HCC, particularly those who do not have significant underlying liver disease. Liver transplantation is also a viable option for patients who meet specific criteria, such as the Milan criteria, which assess tumor size and number, as well as liver function [99,101]. However, these options often yield limited benefits, particularly in advanced disease stages, necessitating the exploration of novel therapeutic approaches and combinations to enhance patient outcomes.

Recent investigations have found that PDE4A expression is considerably elevated in HCC tissues compared to adjacent non-cancerous tissues. This upregulation is associated with poor clinical outcomes, suggesting that PDE4A might act as a prognostic indicator for HCC patients after hepatectomy. The enforced expression of PDE4A in Huh7 cells has been shown to play a significant role in the malignant behavior by inducing epithelial-mesenchymal transition (EMT) [102]. In another study, the autophagic degradation of PDE4A has been shown to activate cAMP/PKA signaling pathways, leading to increased phosphorylation of CREB in the context of HCC. This activation is associated with enhanced expression of TGF-β1, which is known to promote EMT and invasion of cancer cells [89]. PDE4B has emerged as a significant player in various aspects of cancer biology, particularly in the diagnosis, classification, treatment, and prognosis of different malignancies, including bladder cancer, gastric cancer, and prostate cancer, among others [103,104,105,106]. The regulation of PDE4C has been implicated in the modulation of immune responses and the progression of various cancers, suggesting that selective inhibition of this enzyme could provide a novel approach to treatment. The limited availability of selective inhibitors for PDE4C has hindered research into its specific functions and therapeutic potential. However, advancements in drug design and a better understanding of the molecular biology of PDE4C could pave the way for the development of targeted therapies that exploit its unique properties [107]. Elevated expression of PDE4D has been associated with poor survival outcomes in HCC patients. Moreover, PDE4D has been shown to interact with YAP, a key regulator in the Hippo signaling pathway, which is crucial for controlling cell proliferation and survival. The binding of PDE4D to YAP enhances YAP’s dephosphorylation and activity, further promoting HCC cell growth both in vitro and in vivo. This interaction not only underscores the importance of PDE4D in HCC progression but also suggests that targeting the PDE4D-YAP axis with roflumilast could be a viable therapeutic strategy for improving patient outcomes [37].

One study demonstrated that treatment with PDE4 inhibitors, specifically rolipram and DC-TA-46, resulted in a significant increase in intracellular cAMP levels. This increase was associated with a dose- and time-dependent reduction in cell proliferation of HepG2 cells. The inhibitors were found to decrease the expression of cyclins, particularly cyclin A, while simultaneously increasing the levels of cell cycle inhibitors such as p21 and p27. Moreover, the effects of PDE4 inhibitors extend beyond mere cell cycle arrest; they also induce apoptosis in HepG2 cells. This was evidenced by the activation of caspase-3/7 and the occurrence of morphological changes indicative of programmed cell death. The study concluded that PDE4 inhibitors could serve as potential adjuvant therapies in the treatment of HCC, highlighting their dual role in both inhibiting cell proliferation and promoting apoptosis [90]. Zardaverine, a selective inhibitor of PDE3 and PDE4, has shown promising antitumor activity against HCC. The underlying mechanisms of its efficacy may be linked to its regulatory effects on the retinoblastoma (Rb) protein, a critical tumor suppressor involved in cell cycle control [91] (Table 1).

#### 4.5.2. Colorectal Cancer

Colorectal cancer (CRC) is a significant public health concern, being the third most commonly diagnosed cancer and the second leading cause of cancer-related deaths globally. The incidence of CRC has been rising, particularly in low- and middle-income countries, where lifestyle changes associated with westernization, such as increased obesity and sedentary behavior, are contributing factors [92,108]. Despite advancements in treatment options, including surgical interventions, chemotherapy, and targeted therapies, the prognosis for patients with advanced CRC remains poor, particularly for those with metastatic disease [109,110].

Several studies indicate that PDE4 may be an effective target or marker in CRC [111,112,113]. In a three-dimensional colonic crypt model, the expression levels of PDE4B were found to be elevated in clinical tumor samples from CRC patients compared to those from healthy controls. The inhibition of PDE4 catalytic activity using rolipram has demonstrated promising results in reverting the disorganization of cancer cells back to a more physiologically normal state. This reorganization is characterized by the apical assembly of tight junction markers such as ZO-1 and E-cadherin, alongside an increase in apoptosis markers like caspase-3, suggesting that targeting PDE4B could be a viable therapeutic strategy in KRAS-driven CRC [93]. Recent studies have demonstrated that inhibition of PDE4D with rolipram or roflumilast can decrease the malignant properties of colorectal cancer cells by repressing the AKT/mTOR/Myc signaling pathway, which is vital for tumor growth and survival. Specifically, the suppression of PDE4D activity leads to increased intracellular cAMP levels, resulting in reduced oncogenic properties such as colony formation and cell proliferation in DLD-1 CRC cells [94] (Table 1). In another study, the inactivation of oncogenic PDE4D by miR-139-5p in response to p53 activation represents a critical mechanism in CRC biology [114].

In particular, research has shown that GNAS-mutated HCT116 cells exhibit increased cAMP synthesis compared to their parental counterparts. This elevation in cAMP levels correlates with the upregulation of PDE4D, a cAMP-hydrolyzing enzyme, which has been identified as a critical player in the proliferation dynamics of CRC cells. Notably, the expression of PDE4D was validated through RNA sequencing and further confirmed in human CRC tumor samples, indicating a robust association between GNAS mutations and PDE4D overexpression in CRC. The use of PDE4 inhibitors, such as Ro20-1724 and GEBR-7b, has demonstrated a capacity to further suppress the proliferation of GNAS-mutated cells without affecting the parental cell lines, highlighting the potential therapeutic avenues that target PDE4D in GNAS-mutated CRC [115]. Recent studies have shown that PDE4D is a key metastasis-driving target for promoting metachronous metastasis of colorectal cancer through the HIF-1α-CCN2 pathway. The newly synthesized compound L11 can specifically inhibit PDE4D and eliminate metachronous metastasis of colorectal cancer without obvious toxic and side effects [116]. In summary, by targeting PDE4, it may be possible to disrupt critical signaling pathways that promote tumor growth and invasion.

#### 4.5.3. Gastric Cancer

In recent years, PDE4 has emerged as a significant player in the context of GC. Specifically, PDE4B has been shown to promote the progression of GC via the PI3K/AKT/MYC signaling pathway, enhancing cell proliferation and migration while facilitating immune cell infiltration into the TME [105]. Research indicates that the loss of miR-26b-5p can lead to enhanced inflammation-related GC progression, primarily through the activation of a feedback loop involving PDE4B, CDK8, and STAT3 signaling pathways [117]. Targeting this pathway may offer new therapeutic strategies for managing GC, particularly in cases where PDE4B is overexpressed. Further research is warranted to explore the potential of miR-26b-5p as a therapeutic agent and to elucidate the broader implications of PDE4B modulation in GC treatment.

Further investigations into the functional implications of PDE4D have revealed that its depletion can induce apoptosis and inhibit growth across multiple cancer cell types, including gastric, breast, lung, and ovarian cancers. This antitumor effect appears to be lineage-dependent, as it is associated with the induction of pro-apoptotic factors and the downregulation of oncogenic transcription factors. Interestingly, the short isoform of PDE4D, PDE4D2, has been shown to enhance cancer cell proliferation, underscoring the dual role that different isoforms of PDE4D may play in tumorigenesis. In particular, the PDE4D inhibitor 26B has been demonstrated to induce apoptosis and inhibit growth in multiple cancer cell lines [118].

Above all, PDE4 inhibitors represent a novel class of compounds with potential therapeutic applications in the treatment of digestive system tumors. Their ability to modulate cAMP signaling pathways offers a promising avenue for the development of targeted cancer therapies. However, the clinical translation of these inhibitors is hindered by side effects associated with non-selective inhibition. Future research should focus on the development of isoform-specific inhibitors and combination therapies to maximize the therapeutic potential of PDE4 inhibitors in digestive-system oncology.

## 5. PDE4 Inhibitors: Current Status and Challenges

### 5.1. Approved PDE4 Inhibitors

PDE4 inhibitors have emerged as significant therapeutic agents in the management of various inflammatory conditions, with several of them receiving approval for clinical use. Among these, roflumilast, apremilast, and crisaborole stand out due to their distinct mechanisms of action and therapeutic applications. Roflumilast, for instance, is primarily indicated for COPD and has demonstrated efficacy in improving lung function and reducing exacerbation rates in patients suffering from this debilitating condition. Its unique mode of action targets the inflammatory processes underlying COPD, making it a valuable addition to existing treatment regimens [5,119]. In addition to its use in COPD, roflumilast has also been explored as a treatment option for plaque psoriasis, a chronic inflammatory skin condition [120].

Apremilast, another PDE4 inhibitor, has gained attention for its role in treating psoriatic arthritis and plaque psoriasis, and later for oral ulcers of Behcet’s disease. Apremilast has been recognized for its ability to modulate inflammatory pathways by increasing intracellular cAMP levels, which in turn reduces the production of pro-inflammatory cytokines such as TNF-α, IL-12, IL-17, and IL-23, while enhancing anti-inflammatory mediators like IL-10 [121,122,123]. Clinical trials have shown that apremilast can significantly reduce the severity of moderate-to-severe plaque psoriasis, and it is well-tolerated by patients, with mild GI side effects being the most common [124,125].

Crisaborole, the third PDE4 inhibitor, is indicated for topical treatment of atopic dermatitis. Its mechanism involves increasing intracellular cAMP levels, and in turn, this increase enhances anti-inflammatory responses while reducing the production of inflammatory mediators. This makes crisaborole particularly useful for patients with atopic dermatitis who require a localized treatment option with a lower risk of systemic side effects compared to traditional therapies [126].

**Figure 3:**
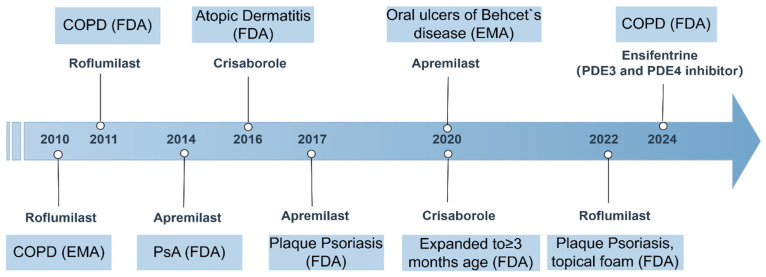
Timeline of PDE4 inhibitor approvals and key indications (2010–2024). This figure summarizes the regulatory approval timeline and major indications for marketed PDE4 inhibitors. COPD, chronic obstructive pulmonary disease; FDA, U.S. Food and Drug Administration; EMA, European Medicines Agency; PsA, psoriatic arthritis.

Ensifentrine, an inhaled selective inhibitor of PDE3 and PDE4, is being developed by Verona Pharma plc for the treatment of respiratory diseases, including COPD. In June 2024, the inhalation suspension of ensifentrine (OHTUVAYRE™) received approval for the maintenance treatment of COPD in adult patients in the United States [127] (Figure 3).

### 5.2. Clinical Evidence

Numerous PDE4 inhibitors are currently in various stages of clinical development for diseases affecting the digestive system. The clinical efficacy, safety and tolerability of apremilast in participants with active UC has been evaluated (NCT02289417). Furthermore, roflumilast is undergoing phase 4 clinical trials for UC (NCT05684484) and has successfully completed a phase 2 clinical trial for NASH (NCT06677788), indicating that its potential in the treatment of digestive diseases is being actively explored. Recently, the safety and efficacy of cilostazol in the treatment of patients with fatty liver disease is being evaluated in a phase 1/2 clinical trial (NCT04761848) (https://clinicaltrials.gov/). Clinical trials of other PDE4 inhibitors in GI and liver diseases are summarized in the table below (Table 2). The data were collected from ClinicalTrials.gov.

### 5.3. Safety and Tolerability

A significant challenge in the development of PDE4 inhibitors is their safety profile. GI side effects and headaches are common, limiting their clinical application. Strategies to improve safety include the development of subtype-selective PDE4 inhibitors and localized administration to reduce systemic exposure. In a recent study, a fusogenic lipid vesicle (FLV) drug delivery system was developed which targets the liver to avoid adverse events. They found that a moderate dose of FLVs-Rol was able to decrease PDE4 activity in the liver without affecting the brain [128].

While PDE4 inhibitors hold therapeutic promise, their safety profile demands careful consideration. Hepatotoxicity, though uncommon, necessitates baseline and periodic liver-function monitoring, particularly in patients with metabolic comorbidities. Drug interactions mediated by cytochrome P450 3A4 (CYP3A4) underscore the need for personalized dosing regimens. CYP3A4-mediated metabolism of PDE4 inhibitors requires dose adjustments when co-administered with inducers or inhibitors of this enzyme. Furthermore, the immunosuppressive effects of pan-PDE4 inhibitors, while beneficial in autoimmune diseases, may predispose patients to opportunistic infections, warranting prophylactic measures in high-risk cohorts. Emerging subtype-selective agents and tissue-targeted delivery systems offer strategies to dissociate efficacy from systemic toxicity, representing a paradigm shift in PDE4-targeted therapies.

## 6. Future Perspectives

Research is focused on identifying more efficient and safer PDE4 inhibitors through novel medicinal chemistry strategies. This includes the development of dual PDE3/4 inhibitors, which may offer improved safety and efficacy profiles. The understanding of PDE4 isoform-specific roles in disease pathogenesis may lead to personalized medicine approaches, where PDE4 inhibitors are tailored to individual patient needs based on their genetic and molecular profiles. Combining PDE4 inhibitors with other therapeutic modalities, such as biologics or immunosuppressants, may enhance their efficacy and reduce the required dosage, thereby minimizing side effects. The combination of PDE4 inhibitors with other therapeutic agents has shown synergistic effects in the treatment of inflammatory diseases and may have potential in the management of digestive system tumors. Future research will focus on optimizing drug formulations to minimize side effects, developing liver-specific delivery systems, and identifying patient populations that would benefit most from PDE4 inhibition.

PDE4 inhibitors represent a novel therapeutic approach for treating inflammatory GI and liver diseases. Significant progress has been made in understanding their mechanisms and clinical applications, with several PDE4 inhibitors already showing promise in early-stage clinical trials. However, challenges related to side effects, targeted delivery, and tissue-specific actions remain. As research continues, there is hope that PDE4 inhibitors will become an essential tool in managing chronic inflammatory diseases of the GI tract and liver, improving outcomes for patients suffering from conditions such as IBD, NAFLD, and NASH. Despite the challenges in their development, ongoing research and clinical trials continue to explore their potential. The future of PDE4 inhibitor therapy lies in the refinement of drug design, a deeper understanding of PDE4 biology, and the development of strategies to minimize side effects while maximizing therapeutic benefits.

## Figures and Tables

**Table 1:** Preclinical evidence of PDE4 inhibitors in GI and liver disease.

PDE4 Inhibitor	Disease/Target	Role	References
Apremilast	DSS-induced colitis; PDE4	Regulates intestinal inflammation, rebuilds the mucosal homeostasis, and remaps gut microbiota	[25,26]
22d	DSS-induced IBD; PDE4, PDE7	Relives inflammatory injuries, and increases body weight	[45]
LZ-14	IBD; PDE4D7	Improves the inflammatory response and colon injury	[46]
Fucoidan	UC; AHR, PDE4	Restores the normal weight and length of the colon; enhances antioxidant activity; modulates gut microbiota; anti-inflammation	[47,48]
HS-1	DSS-induced colitis; PDE4D	Protects the integrity of intestinal epithelial barrier and reduces tissue fibrosis; anti-inflammation	[49]
MnO_2_ and roflumilast-loaded probiotic membrane vesicles	DSS-induced colitis; PDE4	Regulate gut microbe; produce more cAMP and less TNF-α in macrophage	[50]
Roflumilast	NAFLD; PDE4, PDE4D	Reduces hepatic steatosis and fibrosis; improves glucose metabolism	[58,59,60,61,63]
A33 and MDL3	Chronic liver injury and metabolic diseases; PDE4B and PDE5A	Ameliorate pathophysiological signs and symptoms of liver injury and inflammation	[62]
Rolipram	ALD; PDE4, PDE4B	Regulates FA oxidation, and regulates ER stress and apoptosis	[72,73,75]
Apremilast	Alcohol-use disorders; PDE4	Suppresses alcohol intake	[76]
ZL40	ALD; PDE4	Attenuates inflammation and decreases alcohol intake	[77]
KVA-D88-loaded NPs	ALD; PDE4B	Ameliorate alcohol-induced hepatic injury, steatosis, and inflammation	[78]
Rolipram	Bile duct ligation-induced hepatic injury and fibrogenesis; PDE4	Hepatic inflammatory and profibrotic cytokine expression, injury, and fibrosis	[85]
Rolipram	cirrhotic rats with ascites	Increases sodium and phosphate excretion	[86]
Roflumilast	DEN-induced liver fibrosis; PDE4	Modulates cAMP/CREB/TLR4 inflammatory and fibrogenic pathways	[87]
Roflumilast	HCC; PDE4	Inhibits growth, EMT, and invasion of HCC cancer cells	[37,89]
Rolipram and DC-TA-46	HCC; PDE4	Affect HepG2 cell cycle and survival	[90]
Zardaverine	HCC; PDE3/4	Regulates of Rb or Rb-associated signaling in cell cycles	[91]
Resveratrol	CRC; PDE4	Suppresses tumor	[92]
Rolipram	CRC	Disrupts luminal cavity formation and CRC development	[93,94]

ALD, alcoholic liver disease; CRC, colorectal cancer; DEN, diethylnitrosamine; DSS, dextran sulfate sodium; EMT, epithelial–mesenchymal transition; HCC, hepatocellular carcinoma; IBD, inflammatory bowel diseases; UC, ulcerative colitis.

**Table 2:** Clinical trials of PDE4 inhibitors in GI and liver diseases.

Drug Name	Sponsor	ID/Status	Indications	Phase
ASP9831	Astellas Pharma Inc. (Tokyo, Japan)	NCT00668070; completed	NASH	Phase 2
Roflumilast	AstraZeneca (Cambridge, UK)	NCT01703260; terminated	NASH	Phase 2
Roflumilast	Tanta University	NCT06677788; completed	NASH	Phase 2
Roflumilast	Tanta University	NCT05684484; not recruiting	UC	Phase 4
PALI-2108	Palisade Bio (Carlsbad, CA, USA)	NCT06663605; recruiting	UC	Phase 1
Cilostazol	Sadat City University	NCT04761848; active, not recruiting	Fatty liver disease	Phase1/2
Hemay005	Ganzhou Hemay Pharmaceutical Co., Ltd. (Ganzhou, China)	NCT05486104; recruiting	Moderate-to-severe UC	Phase 2
Tetomilast (OPC-6535)	Otsuka Pharmaceutical Co., Ltd. (Tokyo, Japan)	NCT00989573; completed	CD	Phase 3
Tetomilast (OPC-6535)	Otsuka Pharmaceutical Development & Commercialization, Inc. (Rockville, MD, USA)	NCT00064454; completed	UC	Phase 3
OPC-6535	Otsuka Pharmaceutical Co., Ltd.	NCT00317369; terminated	CD	Phase 2
OPC-6535	Otsuka Pharmaceutical Co., Ltd.	NCT00317356; terminated	UC	Phase 2
OPC-6535	Otsuka Pharmaceutical Development & Commercialization, Inc.	NCT00064441; completed	UC	Phase 3
OPC-6535 With Asacol^®^	Otsuka Pharmaceutical Development & Commercialization, Inc.	NCT00092508; completed	UC	Phase 3
Apremilast	Amgen (Thousand Oaks, CA, USA)	NCT02289417; completed	UC	Phase 2

CD, Crohn’s disease; NASH, nonalcoholic steatohepatitis; UC, ulcerative colitis.

## Abbreviations

ACs, adenylate cyclases; AHR, aryl hydrocarbon receptor; ALD, alcoholic liver disease; ALT, alanine aminotransferase; AST, aspartate aminotransferase; AUDs, alcohol-use disorders; BCR, B-cell receptor; cAMP, cyclic adenosine monophosphate; CD, Crohn’s disease; CFTR, cystic fibrosis transmembrane conductance regulator; CLD, chronic liver disease; COPD, chronic obstructive pulmonary disease; COX-2, cyclooxygenase-2; CRC, colorectal cancer; CREB, cAMP response element-binding protein; CYP3A4, cytochrome P450 3A4; DEN, diethylnitrosamine; DSS, dextran sulfate sodium; ECM, extracellular matrix; EMT, epithelial-mesenchymal transition; eNOS, endothelial nitric oxide synthase; EPAC, exchange protein directly activated by cAMP; ER, endoplasmic reticulum; FA, fatty acid; FLV, fusogenic lipid vesicle; GC, gastric cancer; GPCRs, G protein-coupled receptors; HCC, hepatocellular carcinoma; HCN, hyperpolarizing cyclic nucleotide-gated; HS-1, Hypersampsonone H; HSCs, hepatic stellate cells; HUVECs, human umbilical vein endothelial cells; IBD, inflammatory bowel diseases; IκB, inhibitor of NF-κB; iNOS, inducible nitric oxide synthase; LPS, lipopolysaccharide; miRNAs, microRNAs; MnO_2_, manganese dioxide; NAFL, nonalcoholic fatty liver; NAFLD, nonalcoholic fatty liver disease; NASH, nonalcoholic steatohepatitis; NO, nitric oxide; PDE4, phosphodiesterase 4; PDE, phosphodiesterase; PKA, protein kinase A; POPDC, Popeye domain containing; PPARα, peroxisome proliferator-activated receptor alpha; Rb, retinoblastoma; TACE, transarterial chemoembolization; TGF, transforming growth factor; TME, tumor microenvironment; UC, ulcerative colitis; UCRs, upstream conserved regions; YAP, yes-associated protein.
